# Removal of free light chains in hemodialysis patients without multiple myeloma: a crossover comparison of three different dialyzers

**DOI:** 10.1186/s12882-016-0405-5

**Published:** 2016-11-25

**Authors:** Gabriele Donati, Maria Ilaria Moretti, Olga Baraldi, Alessandra Spazzoli, Irene Capelli, Giorgia Comai, Antonio Marchetti, Maria Sarma, Rita Mancini, Gaetano La Manna

**Affiliations:** 1 Nephrology Dialysis and Renal Transplantation Unit, S.Orsola University Hospital, Via Massarenti 9, 40138 Bologna, Italy; 2 Department of Medicine and Public Heath, University of Bologna, Bologna, Italy; 3 Central Laboratory, S.Orsola University Hospital, Bologna, Italy

**Keywords:** PMMA, On-line HFR, Free light chains, Toxins, Removal, Hemodialysis

## Abstract

**Background:**

Immunoglobulin light chains are classified as middle molecule uremic toxins able to interact with B lymphocyte membranes leading to the activation of transmembrane signaling. The ensuing impairment of neutrophil function can contribute to the chronic inflammation state of uremic patients, and the increased risk of bacterial infections or vascular calcifications. The aim of this crossover observational study was to assess the difference in free light chain removal by three different hemodialysis filters in patients not affected by multiple myeloma.

**Methods:**

Free light chain removal was compared in the polymethylmethacrylate (PMMA) membrane Filtryzer BK-F, the polyphenylene HFR17 filter and the conventional polysulfone filter F7HPS. Twenty chronic hemodialysis patients were enrolled: mean age was 67.7 ± 17.0 years, M/F = 14/6, dialysis vintage (months) 25.5 ± 32.0. The patients were randomized into two groups of treatment lasting 6 weeks each. The dialysis sessions checked were the midweek sessions and the blood was drawn at times 0, 120’ and 240’. Kappa (k) and lambda (λ) light chain levels, β2microglobulin (β2M), C reactive protein (CRP) and albumin were checked.

**Results:**

K light chain levels were 345.0 ± 100.0 mg/L, λ light chains were 121.4 ± 27.0 mg/L. The values of k light chains at times 120’ and 240’ were significantly lower with PMMA and HFR17 than those obtained with F7. The reduction ratio per session (RRs) for k light chains was 44.1 ± 4.3% with HFR17, 55.3 ± 3.4% with PMMA, 25.7 ± 8.3% with F7 (*p* = 0.018). The RRs for λ light chains was 30.3 ± 2.9% with HFR17, 37.8 ± 17.3% with PMMA, 14.0 ± 3.9% with F7 (*p* = 0.032). As to β2M, RRs was 42.4 ± 3.2% with HFR17 vs. 33.9 ± 2.8% with PMMA vs. 6.3 ± 1.9% with F7 (*p* = 0.022). The three filters tested showed no differences in CRP or albumin levels.

**Conclusion:**

In terms of light chain and β2M removal, the PMMA and on-line HFR filters are similar and both are significantly more effective than the F7 filter in chronic dialysis patients.

**Trial registration:**

The present trial was registered retrospectively (NCT02950389, 31/10/2016).

## Background

Immunoglobulin light chains are classified as middle molecule uremic toxins together with β2M and parathyroid hormone [1]. They have a mean molecular weight of 25,000 daltons for monomers (k free light chains) and approximately 50,000 daltons for dimers (λ free light chains) [1].

Raised serum levels of polyclonal free light chains can impair neutrophil function in terms of: 1) inhibited chemotactic movement; 2) reduced activation of glucose uptake; 3) inhibited apoptosis [2].

Immunoglobulin light chains are able to interact with B lymphocyte membranes leading to the activation of transmembrane signaling [2]. The ensuing impairment of neutrophil function can contribute to the chronic inflammation state of uremic patients and to the increased risk of bacterial infections [2]. Desjardins et al. demonstrated an association between free light chain levels and vascular calcification progression in chronic kidney disease patients [3]. Nonetheless, serum free light chains in patients with chronic kidney disease are associated with the risk of end stage renal disease and death [4].

It seems that high flux PMMA membranes significantly reduce the light chain level, presumably due to adsorption [5]. This behavior has led to the use of PMMA in the removal of monoclonal free light chains during multiple myeloma [6].

Preliminary reports describe a significant reduction in free light chains also during hemodiafiltration with reinfusion of endogenous ultrafiltrate (on-line HFR) [7]. A recent paper by Borrelli et al. reported an improvement in chronic inflammation after chronic online HFR in dialysis patients [8].

The aim of this crossover observational study was to compare free light chain removal by three different hemodialysis filters in a cohort of chronic dialysis patients not affected by multiple myeloma.

## Methods

One hundred and sixty-three patients on chronic hemodialysis at the Nephrology, Dialysis and Transplantation Unit of S.Orsola University Hospital in Bologna were considered for the study. The patients were enrolled between November 2013 and March 2015.

The inclusion criteria were free light chain values >100 mg/L for k chains and >50 mg/L for λ chains. These levels were chosen arbitrarily because no cut-off levels are available in the literature for the assessment of lowered serum free light chain values during hemodialysis in patients with end-stage renal disease. Fifty-five patients who had light chain levels higher than those required by the inclusion criteria were selected. Among these, 35 patients were excluded: 21 for intradialytic hypotension during bicarbonate dialysis, six patients for multiple myeloma requiring the double PMMA filter application, five for poly-allergy, one for transferral to a peripheral dialysis facility, one for recovery of renal function and one for HIV positivity. Other inclusion criteria were: age >18 years, absence or <200 ml/die residual diuresis, fistula or central venous catheter with blood flow >250 ml/min. Finally 20 patients were enrolled. Patient characteristics are summarized in Table 1. The filters used during the study were: i) HFR17 (Bellco, Mirandola, Italy), a double chamber filter used for online HFR. The first part of the filter consisted in a polyphenylene high flux hemofilter with an ultrafiltration coefficient (Kuf) of 28 ml/h/mmHg, a surface area of 0.7 m^2^ and a membrane cut-off value of 35,000 daltons. The endogenous ultrafiltrate rate is obtained automatically by means of the transmembrane pressure levels in the hemofilter. These are calculated from two pressure sensors: the first is on the arterial bubble chamber and the second before the roller pump of the ultrafiltrate. The ultrafiltrate is driven from this hemofilter to a 40 g neutral styrenic resin that allows an adsorbing area of 28,000 m^2^. After adsorption, the ultrafiltrate is added to the whole blood that, in turn, passes into the second HFR17 filter, a polyphenylene low flux filter (Kuf 13 ml/h/mmHg, surface area 1.7 m^2^) where the weight loss and diffusive depuration take place. ii) PMMA (Toray Filtryzer BK-F, Tokyo, Japan) with a surface area of 2.1 m^2^, a membrane cut-off value of 20,000 daltons and an ultrafiltration coefficient (Kuf) of 26 ml/h/mmHg. iii) A conventional polysulfone membrane (Fresenius F7HPS, Bad Homburg, Germany) with a cut-off of 11,500 daltons, a Kuf of 16 ml/h/mmHg and a surface area of 1.7 m^2^ was used as a control dialyzer.

**Table 1:** Characteristics of the patients enrolled

	Group A	Group B
Age (years)	64.2 ± 16.0	69.8 ± 14.5
Gender (M/F)	7/3	6/4
Dialysis vintage (months)	24.5 ± 30.3	27.2 ± 33.2
Type of dialysis (HD/HDF)	6/4	7/3
Vascular access (CVC/AVF)	8/2	7/3
Kappa light chains (mg/L)	341.3 ± 97.1	353.4 ± 115.5
Lambda light chains (mg/L)	120.8 ± 25.3	124.7 ± 26.2
Serum proteins (g/dl)	5.9 ± 1.1	6.4 ± 0.7
Serum albumin (g/dl)	3.6 ± 0.3	3.5 ± 0.7
Causes of end stage renal disease		
- Nephroangiosclerosis - Type 2 diabetes - Vasculitis - Glomerulonephritis - Polycystic disease - Obstructive nephropathy - Type 1 diabetes - Type 1 oxalosis	4 2 3 0 1 1 0 1	4 1 0 2 0 0 1 0

**Fig. 1:**
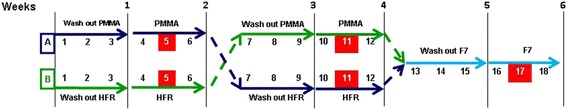
Study design

**Fig. 2:**
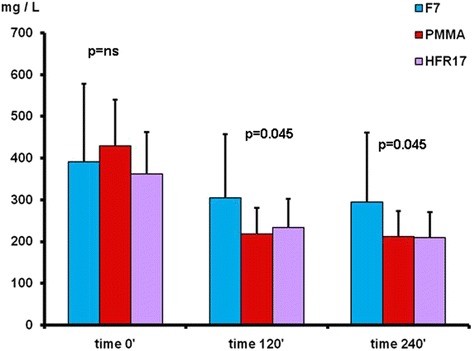
k free light chain removal with the three dialyzers tested at times 120’ PMMA = HFR17 < F7HPS and 240’ PMMA = HFR17 < F7HPS

The patients enrolled were randomized into two groups of treatments lasting 6 weeks each (Fig. 1). Group A: 1st and 2nd weeks with bicarbonate dialysis and filter PMMA, 3rd and 4th weeks with filter HFR17, 5th and 6th weeks with bicarbonate dialysis filter F7. Group B: 1st and 2nd weeks with filter HFR17, 3rd and 4th weeks with bicarbonate dialysis and filter PMMA, 5th and 6th weeks with bicarbonate dialysis filter F7 (Fig. 2). The dialysis sessions carried out during weeks 1, 3 and 5 were considered washout sessions between weeks 2, 4 and 6 when the assessment of λ and k light chains, β2M, C reactive protein and albumin was scheduled. Week number 1 was the washout period between the usual dialytic treatment of the patients enrolled and the beginning of the study. The checking dialysis session was the midweek session and the blood was drawn on starting dialysis (time 0), at two hours (time 120’) and at dialysis end (time 240’). All the dialysis sessions lasted four hours. Mean blood flow was 310 ± 30 ml/min, the mean ultrafiltration rate was 700 ± 200 ml/h, during HFR the endogenous ultrafiltrate rate was 2.3 ± 0.4 ml/h. Dialysate flow was 500 ml/min. Low molecular weight heparin enoxaparin (Clexane®, Sanofi, Milan Italy) was used for anticoagulation of the extracorporeal circuit. Doses of 2000 IU (patients <50 kg of body weight), 4000 IU (patients between 50 to 90 kg of body weight) or 6000 IU (patients >90 kg of body weight) were administered in a single bolus on starting dialysis. Fresenius 5008 and Bellco Flexya dialysis machines were used. The concentrations of k and λ light chains and β2M were measured by nephelometry (kit Freelite k/lambda, The Binding Site Group Ltd., Birmingham, UK; IIMAGE/IMMAGE 800 Beckman Coulter instrument, Brea California USA, Beckman Coulter β2M kit). Normal values: k light chains 3.3 -19.9 mg/L, λ light chains 5.7–26.3 mg/L, β2M 0.7–2 mg/L. Molecular weights: k light chains: 22,500 daltons; λ light chains: 45,000 daltons; β2M: 11,818 daltons. The reference range for patients with normal kidney function was considered between 0.26 and 1.65 according to Bourguignon et al. [9]. CRP concentration was measured by turbidimetry (CRPLX, Tina-quant C-Reactive-Protein; Roche/Hitachi 902 analyzer). CRP normal value < 0.8 mg/dl, molecular weight 120,000 daltons. Albumin was assessed using the common laboratory method. The reduction rate per session (RRs) was calculated as follows [10]:

1 $$ \mathrm{R}\mathrm{R}\mathrm{s} = \left({\mathrm{C}}_{\mathrm{pre}} - {\mathrm{C}}_{\mathrm{post}\hbox{-} \mathrm{corr}}\right)/\ {\mathrm{C}}_{\mathrm{pre}}\times 100 $$

where C_pre_ is the predialysis solute level, and C_post-corr_ is the post-dialysis solute concentration. The values measured during dialysis were corrected for hemoconcentration due to the patient’s weight loss assuming a unicompartimental behavior of light chains described by the following formula [11]:

2 $$ {\mathrm{C}}_{\mathrm{post}-\mathrm{corr}} = {\mathrm{C}}_{\mathrm{post}}/\ \left\{1 + \left[\Delta \mathrm{B}\mathrm{W}/\left(0.2\times {\mathrm{BW}}_{\mathrm{post}}\right)\right]\right\} $$

where C_post-corr_ is the post-dialysis solute concentration and C_post-corr_ is the concentration of light chains corrected for the hemoconcentration, ΔBW is the weight subtracted during dialysis, and BW is the body weight at the end of dialysis.

### Statistical analyses

Statistical analysis was performed using SPSS statistical software. The data are presented as mean ± standard error of the mean. The Shapiro-Wilk test, suitable for small populations, showed a non-normal distribution of the variables, for which the non-parametric Friedman test was used. The *p* value <0.05 was considered normal. The Wilcoxon Signed Rank Test was subsequently used to see which pairwise comparisons resulted statistically different. In addition, as a multiple comparison was done, we applied the Bonferroni correction, thereby setting a *p* value = 0.05 / 3 = 0.0167. For each of the sampling times 0.120 and 240 min, we compared the results and the RRs obtained with the different filters (HFR17, PMMA, F7).

## Results

HFR17 removal of free light chains was statistically significant between time 0’ (k: 361.6 ± 100.7 mg/L; λ 122.4 ± 27.6 mg/L) and time 120’ (k: 233.6 ± 68.8 mg/L; λ 84.3 ± 15.6 mg/L) for both k and λ chains (p <0.001). K and λ chain concentrations were reduced between times 120’ and 240’ but the value did not reach statistical significance (*p* = ns). HFR17 significantly reduced β2M values between time 0’ (38.4 ± 6.0 mg/L) and time 120’ (23.9 ± 3.6 mg/L) and between times 120’ and 240’ (at time 240’ β2M values were 21.6 ± 4.1 mg/L) (*p* = 0.04). CRP values did not exhibit statistically significant changes at the three times considered.

PMMA significantly reduced k and λ chains between time 0’ (k: 429.4 ± 110.3 mg/L; λ: 120.1 ± 19.9 mg/L) and time 120’ (k: 218.5 ± 62.2 mg/L; λ: 72.2 ± 14.3 mg/L) (*p* < 0.001), but no significant reduction was found (*p* = ns) between time 120’ and time 240’. A significant reduction was found for β2M between time 0’ (39.3 ± 5.4 mg/L) and time 120’ (27.3 ± 4.0 mg/L) (*p* < 0.001), but not between times 120’ and 240’ (*p* = ns).

The values obtained for the k light chains with the F7 filter showed a non-significant reduction during each time interval considered; the same behavior was found for λ chains and β2M values.

**Fig. 3:**
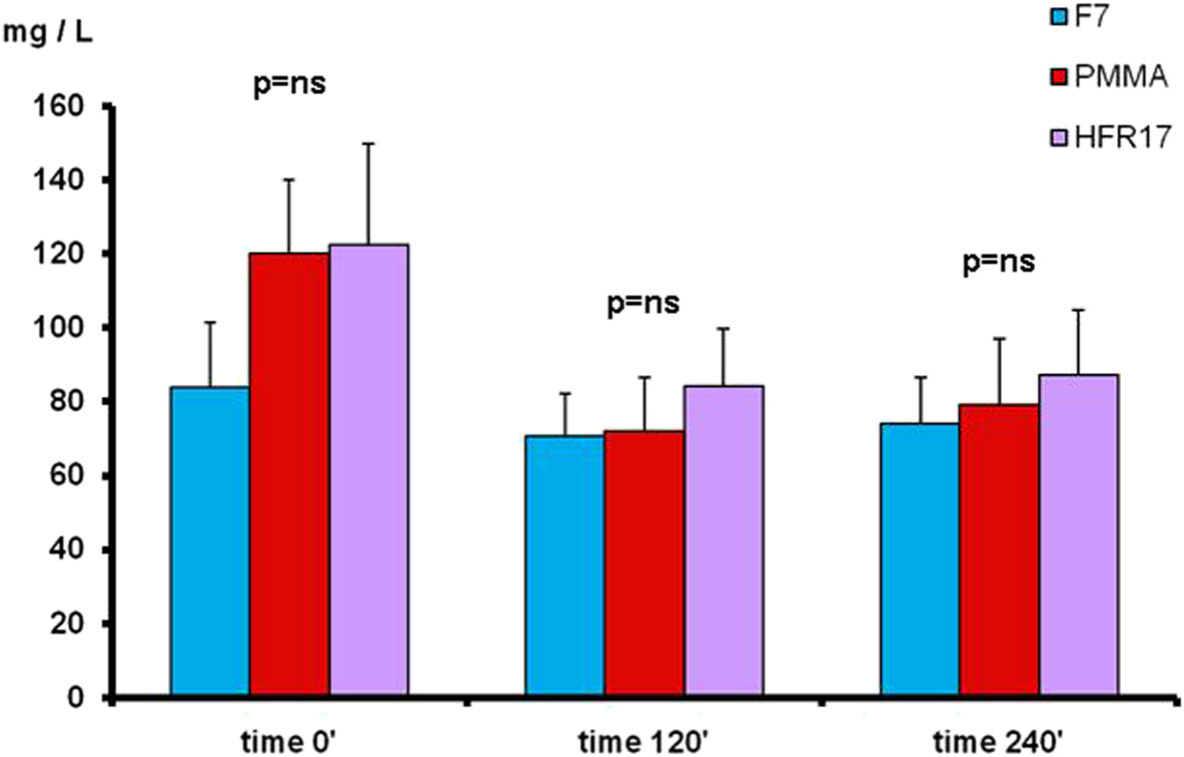
λ free light chain removal with the three dialyzers tested: no differences were found at the checking times considered

**Fig. 4:**
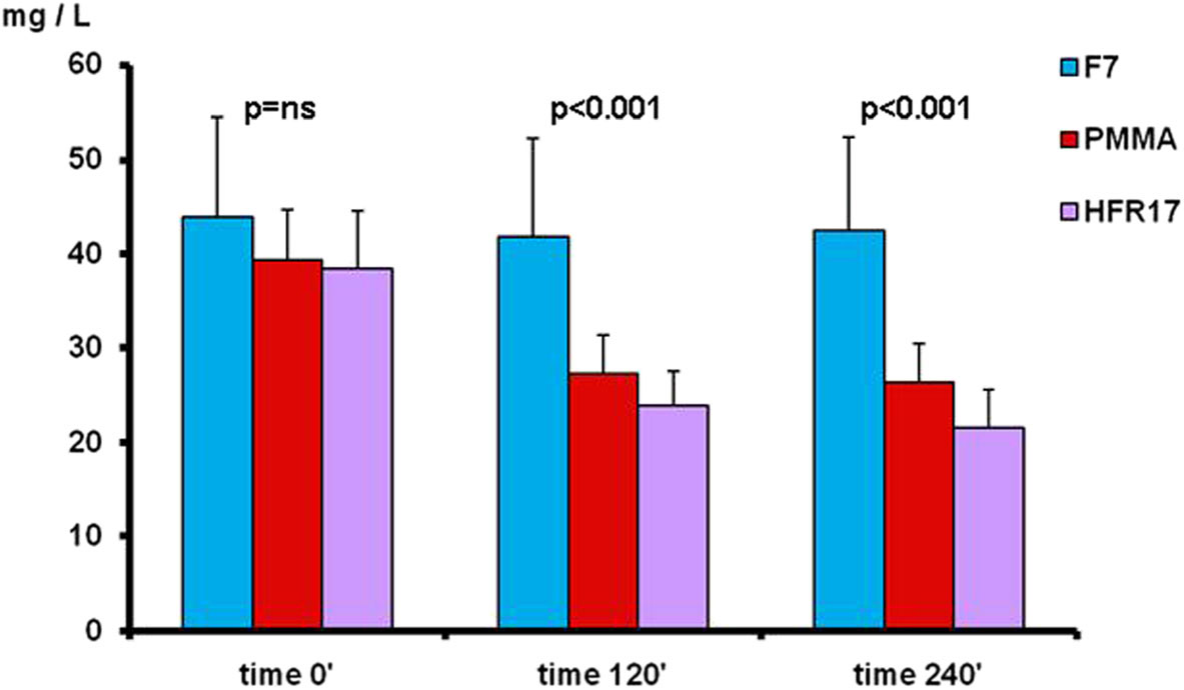
β2M removal with the three dialyzers tested at times 120’ PMMA = HFR17 < F7HPS and 240’ PMMA = HFR17 < F7HPS

**Fig. 5:**
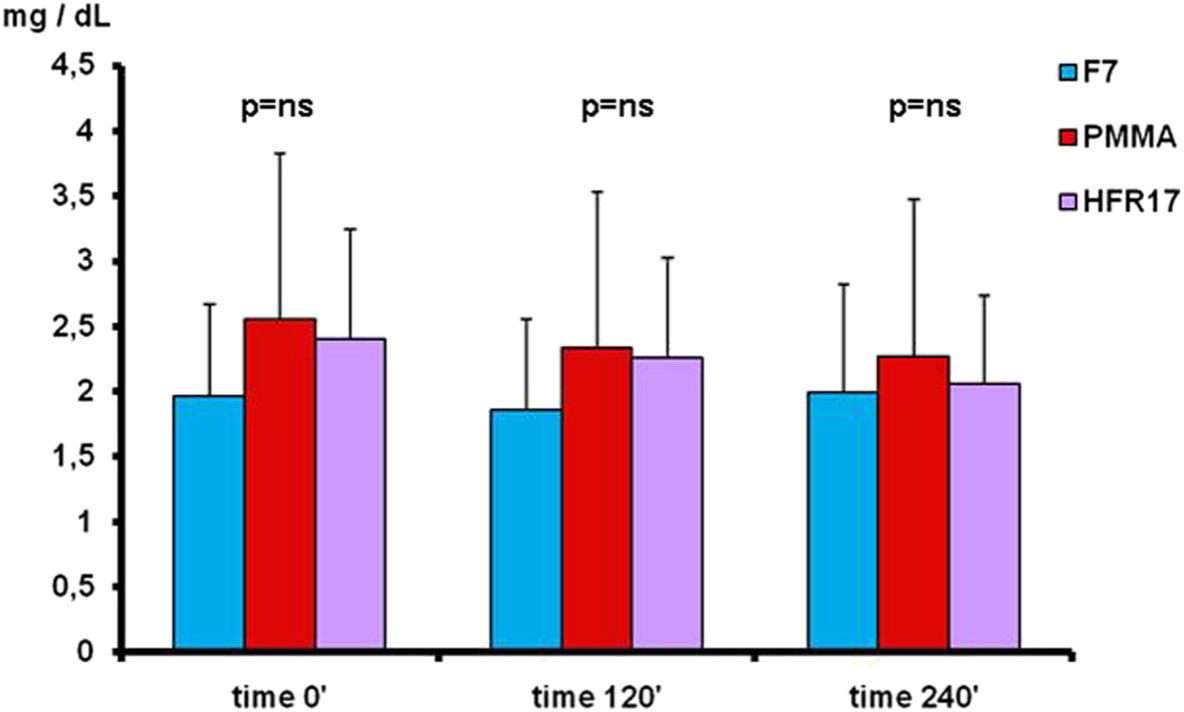
CRP values with the three dialyzers tested at the checking times considered

By comparing the different filters used at the different checking times the following results were obtained (Fig. 2): k light chains levels at time 0’ did not differ among the three filters tested (*p* = ns). At time 120’ the three filters differed significantly with the PMMA filter showing the lowest value (*p* < 0.05). At time 240’ the k light chains level were significantly lower with HFR17 and PMMA in comparison to F7 (*p* < 0.05) (Fig. 2). No difference in λ light chain levels was found among the three filters tested at times 0’, 120’ and 240’ (Fig. 3). B2M levels at time 0’ did not differ among the three filters tested. At time 120’, β2M levels were lower with HFR17 and PMMA in comparison with F7 (*p* < 0.001) and at 240’ (*p* < 0.001) when HFR17β2M levels seemed slightly lower than those of PMMA (Fig. 4). CRP values at times 0’, 120’ and 240’ did not differ among the three filters tested (Fig. 5).

**Fig. 6:**
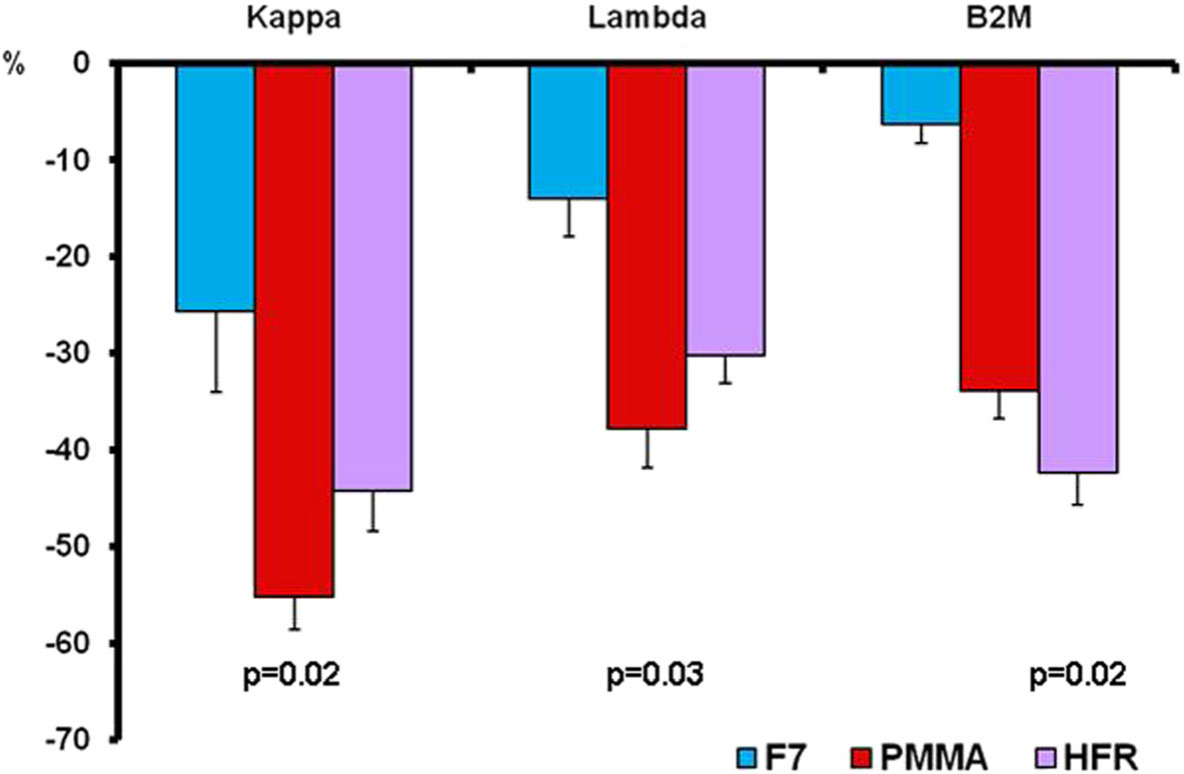
Comparison of the RRs obtained with the three dialyzers tested for k and λ light chains and for β2M. PMMA = HFR17 > F7HPS

Finally the RRs was measured for k and λ light chains and β2M. For k light chains, RRs values were higher with HFR17 and with PMMA than those obtained with F7 (*p* = 0.018). RRs of λ light chains and of β2M showed the same behavior (*p* = 0.032 and *p* = 0.022 respectively) (Fig. 6).

**Fig. 7:**
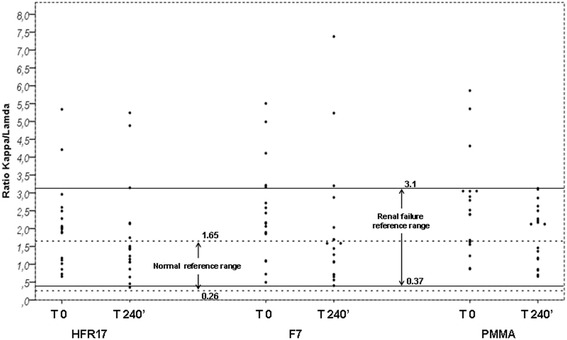
Effect of dialysis with HFR17, PMMA and F7 on k/λ ratio before dialysis (time 0’) and after dialysis (time 240’). Dashed lines = normal reference range. Continuous lines = extended renal failure reference range [9]

Predialysis and postdialysis k/λ ratio values with the three dialyzers tested are showed in Table 2 and Fig. 7. After dialysis, the frequency of patients with a k/λ ratio in the normal range described for subjects with normal kidney function was 60% (12/20) with HFR17, 55% (11/20) with PMMA and 40%(8/20) with F7 (*p* = ns).

**Table 2:** Ratio k/l light chains before (time 0) and after dialysis (time 240’)

	HFR 17	PMMA	F7	P
Ratio k/l light chains				
-time 0	7.7 ± 4.2	8.9 ± 4.7	10.6 ± 6.1	ns
-time 240’	4.8 ± 2.2	6.8 ± 3.6	14.3 ± 10.4	0.02
	0.02	<0.001	ns

There was no difference in albumin levels among the three dialyzers tested. Using HFR17 albumin was 3.6 ± 0.4 g/dl on starting dialysis vs. 3.7 ± 0.7 at dialysis end (*p* = ns). With PMMA albumin values on starting dialysis were 3.6 ± 0.4 g/dl vs. 3.6 ± 0.7 g/dl at dialysis end (*p* = ns). Using the F7HPS dialyzer albumin was 3.6 ± 0.4 g/dl on starting dialysis vs. 3.7 ± 0.6 g/dl at dialysis end (*p* = ns). No difference among the three dialyzers was found comparing the values obtained with each filter at the checking times considered in the study.

Spearman’s correlation test was used to evaluate any correlation between the blood flow rate during the single dialysis session and the k and λ free chain concentration. The correlation coefficient for the PMMA dialyzer at 120’ was 0.056 (*p* = ns) for k light chains, -0.272 (*p* = ns) for λ light chains. At dialysis end it was -0.161 (*p* = ns) for k light chains and -0.131 (*p* = ns) for λ light chains. The correlation coefficient for HFR17 at 120’ was -0.483 (*p* = ns) for k light chains, -0.268 (*p* = ns) for λ light chains. At dialysis end it was -0.161 (*p* = ns) for k light chains and -0.075 (*p* = ns) for λ light chains.

## Discussion

The present study showed that the k light chains were significantly lower with HFR17 and PMMA dialyzers than with the F7 during a four-hour dialysis treatment. Free light chain removal had already approached a plateau at the second hour of dialysis with both PMMA and HFR17 filters. For both PMMA and HFR17 no further significant improvement in k and λ light chains was found in the second part of the dialysis sessions. The RRs showed free k light chain removal was higher with PMMA than with the HFR17 or F7 dialyzers. λ light chain removal peaked after two hours of dialysis treatment but without statistical differences among the three filters. This is probably explained by the small cohort of patients enrolled and by the low levels of λ light chains considered suitable for the study at dialysis start. λ free light chain removal seems to be more difficult due to the dimeric structure and higher molecular weight than k light chains [12]. Nonetheless, PMMA showed significantly higher RRs for λ free light chains than the HFR17 and F7 dialyzers. β2M removal at the second hour of treatment was already significantly lower with both HFR17 and PMMA than with the F7 dialyzer. The β2M RRs was significantly higher with both online HFR and PMMA in comparison with F7.

Many studies have sought to establish the optimal dialysis technique for free light chain removal during multiple myeloma with renal involvement. However, the issue of free light chains as uremic toxins in non-multiple myeloma patients has received only marginal attention [2]. In the field of free light chain removal the PMMA membrane is the first choice technique since this membrane removes proteins not only via permeation but also via an adsorptive mechanism [5]. In 2002, Cohen enrolled 71 non-myeloma chronic hemodialysis patients treated with bicarbonate dialysis and 33 patients treated with hemodiafiltration [13]. He tested free light chain removal by six different dialyzers in the group of patients treated with bicarbonate dialysis, and three dialyzers in the group of patients who underwent hemodiafiltration. Free light chains, namely λ free light chains, were significantly reduced only with the PMMA dialyzer. The removal rate considered two hours after dialysis start was 43% for λ free light chains and 12% for the k free light chains. Unfortunately only four patients underwent PMMA hemodialysis while four patients underwent PMMA hemodiafiltration [13]. Fabbrini et al. carried out a retrospective study on ten acute and chronic dialysis patients with high free light chain levels (k or λ > 500 mg/dl). Five patients underwent bicarbonate dialysis with a single PMMA filter and the other five patients with the “enhancing adsorption properties technique” (EAD) [14]. With EAD the PMMA dialyzer is replaced after two hours of dialysis treatment. The five single PMMA filter patients showed free light chain RRs corresponding to 22.3% for k light chains and 21% for λ light chains after four hours of treatment. The five patients who underwent EAD showed RRs of 31% for k free light chains and 53.1% for λ free light chains [14].

Testa et al. enrolled 11 chronic hemodialysis patients undergoing online HFR, finding a significant reduction of k and λ free light chains with the HFR17 dialyzer. The mean reduction of k light chains was 30% and 20% for λ free light chains [7]. The removal of free light chains during online HFR depends on the adsorption obtained by the resin cartridge of the HFR17 filter. This was confirmed by an in vitro experiment where 10 ml of styrenic neutral resin were incubated with the serum of patients with IgA-k and IgG-k multiple myeloma. The serum monoclonal protein was significantly reduced, but unfortunately their study did not include a control group [7].

A conventional polysulfone membrane like the F7 seems to allow some removal of free light chains by adsorption because its cut-off is about 11,000 daltons and it does not allow free light chain removal by diffusion or convection. Birk et al. observed that one type of polysulfone (Fresenius F60) showed a higher adsorption of both total proteins and low molecular weight proteins than another polysulfone membrane tested (D30) during an “in vitro” comparison of 11 different membrane materials [15]. Interestingly, Lamy et al. enrolled 31 chronic hemodialysis patients in a prospective observational study evaluating the reduction of free light chain values with high-flux dialyzers like FX80 and FX100 [15]. The RRs for k free light chains was 66% with online HDF vs. 52% with bicarbonate dialysis (*p* < 0.001), whereas no difference was found between the two techniques tested for λ free light chains (37% RRs with both techniques) [16].

Our study found no differences in CRP levels among the three dialyzers tested. CRP is considered a marker of intradialytic inflammatory activation and selective solute removal by the dialyzers tested [17]. Another important issue during light chain removal is the albumin loss: filters with a high cut-off allow an albumin loss that can approach 63 g/session [18]. No albumin loss was found during each session with the three dialyzers tested confirming the selectivity of the adsorption. This was due to the low cut-off value of the PMMA and F7 dialyzers and to reinfusion of the endogenous ultrafiltrate after adsorption obtained with the neutral resin of the HFR17 dialyzer into the whole blood returned to the patient.

The main weaknesses of this study are the small cohort of patients enrolled, the low levels of λ light chains considered suitable for the study at dialysis start and the limited number of biomarkers considered. Conversely, one of the strengths of the study is its crossover study design, which allows each patient to be his/ her control, strongly reducing the variability among patients. Other strengths are the randomization of the patients into two groups and the washout periods between the experimental phases with each filter tested, reducing the carryover effect of the previous filter / hemodialysis technique used.

## Conclusions

Our study yielded some important results for everyday clinical practice. Firstly, the routine use of PMMA and HFR17 filters can significantly reduce the burden of free light chains and β2M in chronic dialysis patients not affected by multiple myeloma. PMMA was more effective in removing free light chains, while HFR17 proved more effective in the removal of β2M [19]. Secondly, this efficacy allows the chronic use of the two dialyzers in chronic dialysis patients when the recovery of multiple myeloma is obtained but not the recovery of renal failure. In this case, use of two PMMA filters for each dialysis session or use of the HFR-Supra is not indicated because of the low light chain values [20, 21]. Thirdly, the polysulfone membrane showed a surprising intrinsic adsorptive ability, recently improved by the technical evolution of this membrane as in the case of polyethersulfone and polyester polymer alloy membrane filters [9, 22]. The depurative efficiency of the dialyzers tested on the middle molecules considered should encourage new studies with more patients to confirm these results on a large number of uremic toxins in the middle molecule range.

## Abbreviations

BW: Body weight at the end of dialysis
C_post_: The post-dialysis solute concentration
C_post-corr_: The concentration of light chains corrected for the hemoconcentration
C_pre_: The predialysis solute level
CRP: C reactive protein
EAD: Enhancing adsorption properties technique
g: Grams
k: Kappa
Kuf: Coefficient of ultrafiltration
online HDF: Online hemodiafiltration
online HFR: Hemodiafiltration with reinfusion of the endogenous ultrafiltrate
PMMA: Polymethylmethacrylate
RRs: Reduction rate per session
β2M: β2microglobulin
ΔBW: The weight subtracted during dialysis
λ: Lambda
